# ZILA-SRM: a probabilistic framework with zero-inflated latent models for robust strain reconstruction from metagenomes

**DOI:** 10.1128/spectrum.04101-25

**Published:** 2026-06-30

**Authors:** Saidi Wang, Mintong Chen, Di Jiao

**Affiliations:** 1 School of Artificial Intelligence, Henan University, Zhengzhou, China; 2 Institute for Animal Health, Key Laboratory of Animal Immunology, Henan Academy of Agricultural Sciences, Zhengzhou, China; American Type Culture Collection, Manassas, Virginia, USA

**Keywords:** strain reconstruction, shotgun metagenomic data, metagenomics, zero-inflated Poisson, adaptive sparsity regularization

## Abstract

Resolving bacterial strain diversity from shotgun metagenomic data is fundamental to understanding intra-host evolution, transmission dynamics, and phenotypic heterogeneity. However, current probabilistic approaches face a severe “identifiability limit” when disentangling highly similar genomes. Under high-noise conditions, sequencing errors, coverage overdispersion, and collinearity confound standard expectation-maximization algorithms, resulting in overfitting and spurious “ghost” strains. Here, we introduce zero-inflated latent allocation for strain reconstruction from metagenomes with adaptive sparsity regularization (ZILA-SRM) to overcome this barrier through three innovations. First, we integrate a zero-inflated Poisson mixture model to decouple "structural zeros" (true strain absence) from "sampling zeros" (stochastic dropout), addressing overdispersion in standard Poisson-based tools. Second, we impose a convex adaptive sparsity regularization penalty that leverages biological sparsity priors to shrink noise artifacts dynamically. Third, we implement a graph-theoretic refinement step using maximal clique enumeration to resolve haplotype collinearity. Benchmarking against StrainFinder and MixtureS on 702 synthetic data sets shows that ZILA-SRM achieves a 20% improvement in precision in high-complexity scenarios while maintaining over 80% recall for minor variants at 0.5% abundance. Re-analysis of deep-sequencing data from 195 *Mycobacterium tuberculosis* clinical samples reveals cryptic low-abundance drug-resistant variants in 12% of patients, including a minor clone carrying the rpoB S450L mutation. Furthermore, application to skin microbiome data sets further reveals a strong negative correlation between dominant *Staphylococcus aureus* and *Staphylococcus epidermidis* strains, providing genomic evidence for competitive exclusion. These findings establish ZILA-SRM as a robust tool for resolving strain-level diversity in complex metagenomes.

**IMPORTANCE:**

Understanding microbial communities at the strain level is critical because closely related strains can differ dramatically in traits such as drug resistance, virulence, and ecological interactions. However, resolving individual strains from metagenomic sequencing data remains difficult, especially when strains are highly similar or present at low abundance. As a result, biologically meaningful diversity is often obscured or misinterpreted as noise. In this study, we introduce a new framework that improves the reliability of strain reconstruction from complex metagenomic data. By reducing false-positive strain detection while preserving sensitivity to rare variants, our approach enables more accurate characterization of microbial populations. This improved resolution reveals previously hidden subpopulations in clinical and microbiome datasets, providing clearer insights into microbial evolution, competition, and the emergence of clinically relevant traits such as antibiotic resistance.

## INTRODUCTION

The resolution of metagenomic sequencing has progressively deepened from the phylum to the species level, providing unprecedented insights into the structure and function of microbial communities (1). Yet, the strain level, where critical phenotypic differences such as drug resistance, virulence factors, and metabolic capacities reside, remains the frontier of computational biology (2). While marker-gene approaches can estimate strain presence based on unique clade-specific markers (3), they fundamentally lack the resolution to track transmission pathways, understand durable coexistence, or monitor evolutionary trajectories (4). Whole-genome reconstruction is essential for these tasks, particularly in clinical contexts where distinguishing between a relapse (recrudescence of the same strain) and reinfection (acquisition of a new strain) dictates therapeutic strategy (5).

The biological imperative for strain-level resolution is underscored by the epidemiology of infectious diseases. In *Mycobacterium tuberculosis* (TB), the difference between a drug-susceptible infection and a multi-drug resistant one is often defined by single-nucleotide polymorphisms (SNPs) in specific loci, such as rpoB (6, 7). Recent genomic epidemiological studies in 2024 and 2025 have highlighted that mixed infections and heteroresistance, where susceptible and resistant subpopulations coexist within a host, are far more prevalent than previously thought, often acting as precursors to treatment failure (8–10). Similarly, in the human microbiome, commensal strains of *Staphylococcus epidermidis* may protect against skin pathogens, while other strains of the same species may harbor virulence factors that exacerbate disease states like atopic dermatitis (11). Thus, the “species” designation often masks a vast functional heterogeneity that can only be resolved by reconstructing individual haplotypes.

Currently, computational approaches for strain resolution fall into two broad categories: reference-based profiling and *de novo* model-based reconstruction. Reference-based tools excel at tracking known lineages but are limited by database completeness (12). Conversely, model-based reconstruction algorithms aim to infer strain genomes directly from allele frequency profiles. This category is dominated by methods relying on expectation-maximization frameworks to maximize likelihood, most notably StrainFinder (13) and MixtureS (14). However, recent benchmarking has shown that while long-read sequencing is improving resolution (15), short-read data remain the standard for cost-effective population profiling, necessitating robust algorithms to handle inherent noise (16).

Despite their utility, *de novo* strain reconstruction from short-read data is an ill-posed inverse problem. When multiple strains coexist, assigning reads to specific haplotypes becomes statistically ambiguous, particularly when reference genomes share more than 99% of their sequence (17). In these regimes, the “curse of dimensionality” manifests: as the number of potential strains increases, the solution space expands exponentially while available data (read coverage at polymorphic sites) remain fixed. To rigorously evaluate progress in this domain, we selected StrainFinder (13) and MixtureS (14) as our primary benchmarks. The rationale for this focused selection is threefold. First, StrainFinder represents the foundational “gold standard” for *de novo* reconstruction using maximum likelihood estimation (MLE), establishing the baseline for probabilistic inference. Second, MixtureS represents a more recent advancement that attempts to handle coverage covariance, yet still struggles to distinguish low-abundance variants from sequencing error in high-similarity scenarios (>99.5% average nucleotide identity). Comparing zero-inflated latent allocation for strain reconstruction from metagenomes with adaptive sparsity regularization (ZILA-SRM) against these two defines the exact boundary of the current “identifiability limit.” Thirdly, other tools such as DESMAN (18) or StrainGE (12) either rely on different input data structures (gene presence/absence) or reference-based heuristics that do not address the core mathematical challenge of *de novo* haplotype inference from allele frequencies.

Standard MLE-based approaches suffer from a common pathology: overfitting (19). Faced with coverage heterogeneity (overdispersion) and sequencing errors, these algorithms tend to maximize likelihood by fragmenting coverage into a continuum of “ghost strains” that mathematical artifacts created to explain variance which is actually due to noise. For example, in a sample containing one dominant strain, an unregularized solver might infer three strains to “perfectly” fit the noisy frequency distribution. This creates a “dense fog” of false positives that confounds biological interpretation.

We posit that the failure of current methods is not merely algorithmic but conceptual. Biological systems are inherently sparse. Within a specific ecological niche, competitive exclusion principles suggest that communities are dominated by a finite set of fittest clones rather than an infinite spectrum of variants (20). This principle of sparsity—that the true number of strains is small relative to the number of possible strains—provides a powerful constraint for regularization. To operationalize this, we developed ZILA-SRM. Unlike predecessors, ZILA-SRM introduces three key innovations:

1. Zero-inflated latent allocation (ZILA): a generative model that explicitly captures sequencing dropout by distinguishing “structural zeros” (absence) from “sampling zeros” (dropout) (21).
2. Adaptive sparsity regularization (ASR): a convex optimization strategy that acts as a mathematical “Occam’s Razor,” dynamically penalizing model complexity to shrink the abundance of unsupported artifacts to zero (22).
3. Hybrid graph refinement: a topological post-processing step that resolves collinearity artifacts by merging phylogenetically consistent cliques (23).

## MATERIALS AND METHODS

### Overview of the ZILA-SRM framework

**Fig 1:**
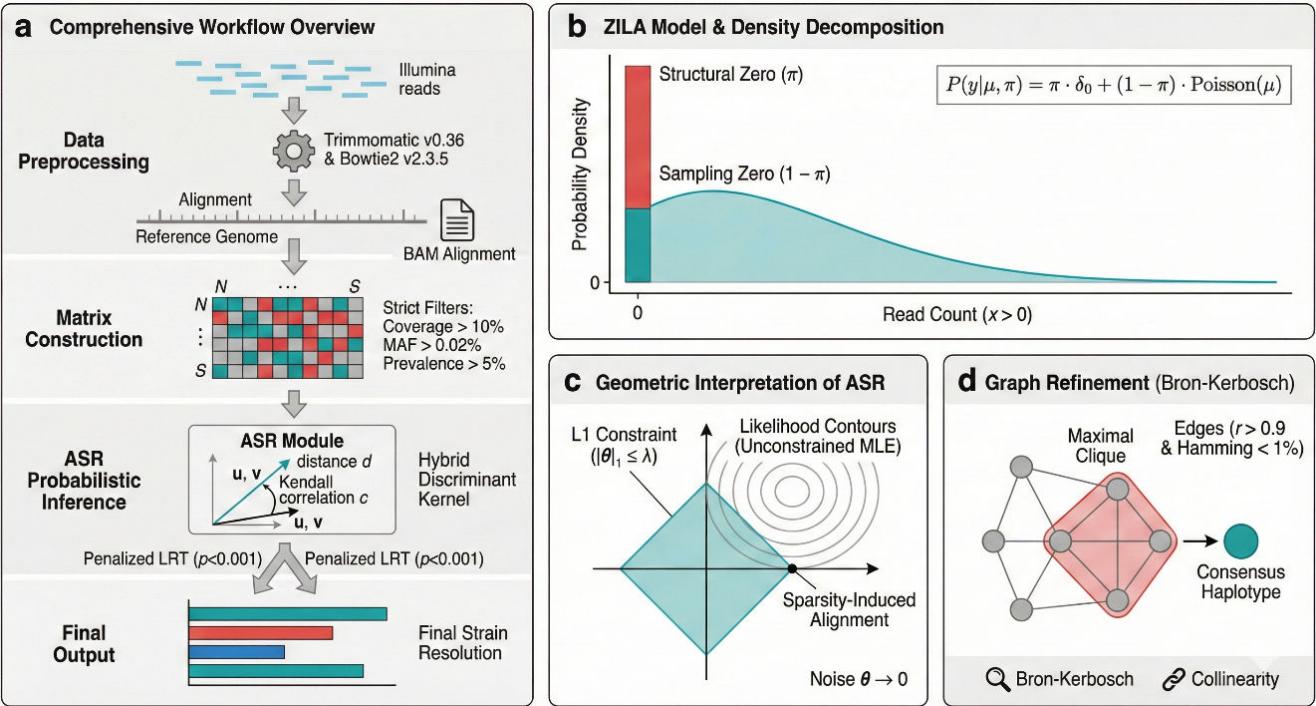
The comprehensive workflow. Panel (**a**) details the preprocessing of raw Illumina reads using Trimmomatic and alignment via Bowtie2 to generate a variant matrix filtered by strict criteria (Coverage>10%, MAF>0.02, Prevalence>5%) to separate signal from noise. Panel (**b**) illustrates the ZILA model density decomposition, distinguishing “Structural Zeros” (π) from “Sampling Zeros” (1−π). Panel (**c**) visualizes the geometric interpretation of the ASR $L_{1}$ constraint ($|\theta {|}_{1}\leq \lambda$), which forces the likelihood maximization solution toward the axes, inducing sparsity. Finally, panel (**d**) depicts the graph refinement step where the Bron-Kerbosch algorithm identifies maximal cliques to merge consistent haplotypes.

ZILA-SRM is designed for the *de novo* reconstruction of bacterial strains from shotgun metagenomes. The workflow is predicated on the axiom that single-nucleotide variants (SNVs) originating from a single lineage exhibit coherent coverage covariance across multiple samples. The pipeline comprises three phases: (i) high-fidelity variant profiling, (ii) probabilistic strain inference via ASR, and (iii) graph-theoretic consistency refinement, as illustrated in Fig. 1.

### Data preprocessing and matrix construction

The foundation of accurate strain reconstruction is high-quality variant calling. Raw sequencing reads were quality-trimmed using Trimmomatic to remove adapter sequences and low-quality bases (Q<20) (24). The trimmed reads were aligned to species-specific reference genomes using Bowtie2 with default parameters (25). To construct the observation matrix $Y\in N^{N\times S}$, where N is the number of SNV sites and S is the number of samples, we identified SNVs satisfying strict criteria designed to filter out sequencing noise while retaining low-abundance variants:

1. Global coverage: the site must have a coverage depth >10% of the average sample coverage. This ensures that the site is well-supported by reads.
2. Minor allele frequency (MAF): >0.02. This threshold excludes singleton errors while retaining sensitivity for minor strains down to 2% abundance.
3. Sample prevalence: >5%. The variant must appear in at least 5% of samples to provide sufficient covariance signal for the probabilistic model.

### The ZILA model

Standard Poisson models used by StrainFinder and MixtureS assume that the variance of the count data equals the mean ($\sigma^{2}=\mu$). However, metagenomic count data typically exhibit overdispersion ($\sigma^{2}>\mu$) due to PCR amplification bias and stochastic dropout (26). A simple Poisson model cannot account for the excess zeros observed in such data, treating them as extremely unlikely events and skewing the parameter estimation to compensate. ZILA-SRM addresses this by formulating a zero-inflated Poisson generative model. We posit that the observed data come from a mixture of two distributions: a point mass at zero (representing “Structural Zeros”) and a Poisson distribution, which can generate both non-zero counts and “Sampling Zeros.”

Let $y_{ij}$ be the observed read count for SNV i in sample j. The probability mass function is given by


$$\begin{array}{r} P(y_{ij}|\mu_{ij},\pi_{j})=\{\pi_{j}+(1-\pi_{j})e^{-\mu_{ij}}\;if\;y_{ij}=0\\ (1-\pi_{j})\frac{e^{-\mu_{ij}}\mu_{ij}^{y_{ij}}}{Y_{ij}!}\;if\;y_{ij}>0 \end{array}$$

Here, $\pi_{j}$ is the zero-inflation parameter (probability of structural absence) for sample j. It captures the probability that the strain is effectively absent or below the detection threshold due to technical reasons unrelated to Poisson sampling. $\mu_{ij}$ is the expected read count, modeled as $\mu_{ij}=D_{j}\sum_{k=1}^{K}\;G_{ik}\theta_{kj}$, where $D_{j}$ is the sequencing depth, $G_{ik}$ is the genotype, and $\theta_{kj}$ is the relative abundance. This decomposition explicitly distinguishes between “Structural Zeros” (strain absence) and “Sampling Zeros” (stochastic dropout). By introducing the latent variable $Z_{ij}$ to track the source of zeros, ZILA allows the algorithm to “trust” non-zero counts in low-coverage regions without being penalized by the abundance of zeros, effectively recovering the signal of rare variants that standard models dismiss as noise.

### Strain inference via ASR

In the unregularized setting (e.g., standard MLE), maximizing likelihood often leads to a solution with non-zero abundances for all potential strains. This is the identifiability limit: the algorithm creates “ghost strains” to explain small fluctuations in coverage. To enforce the biological prior of sparsity, we impose a global constraint using ASR. The objective function J(θ) maximizes the penalized likelihood:


$$J\left(\theta \right)=L_{\text{ZILA}}\left(\theta \right)-\lambda \sum_{k=1}^{K}\;w_{k}|\theta_{k}|$$

where λ is the tuning parameter, and $w_{k}$ are adaptive weights. The $L_{1}$-regularization ($|\theta {|}_{1}$) creates a diamond-shaped constraint region in the parameter space (Fig. 1c). Geometrically, the optimal solution is found where the likelihood contours touch the constraint region. Due to the “pointy” corners of the $L_{1}$ diamond, the solution is forced to occur where coefficients are exactly zero. Unlike static Lasso, ASR weights the penalty for each coefficient by the inverse of an initial consistent estimate ($w_{k}=1/|{\overset{ˆ}{\theta }}_{k}^{\text{init}}{|}^{\gamma }$). This “oracle property” ensures that ZILA-SRM selectively suppresses noise while preserving true low-abundance signals. Figure S1 demonstrates the sensitivity analysis of the regularization parameter λ, showing optimal stability around λ=80 for high-complexity data sets.

### Hybrid graph-theoretic refinement

Standard probabilistic methods often struggle with collinearity, where two distinct strains fluctuate in abundance identically across samples. To resolve this, we implement a *post hoc* graph-theoretic consistency refinement. We construct a consistency graph where nodes are inferred haplotypes and edges represent high consistency. Consistency is defined by a hybrid discriminant kernel combining Euclidean distance and Kendall’s tau rank correlation, which captures ordinal association robust to normalization artifacts (Fig. S5). Edges are drawn if haplotypes have high abundance correlation (r>0.9) and genomic similarity (Hamming distance <1%). We then employ the Bron-Kerbosch algorithm to identify maximal cliques. Strains within a clique are merged into a single consensus haplotype. This topological step eliminates chimeric artifacts and “fragmented” strains, ensuring that the final output represents phylogenetically distinct lineages.

### Simulation benchmarking parameters

We generated 702 synthetic data sets using the DWGSIM (27), a whole-genome simulator for next-generation sequencing, available at https://github.com/nh13/DWGSIM. Data sets were stratified into four complexity levels (Types 1–4) to stress-test the algorithms, as detailed in Table S1. Type 4 represents the “identifiability limit,” where strains share more than 30% to 90% of SNPs. This high-similarity regime effectively mimics the challenge of distinguishing closely related pathogenic clones in a clinical outbreak.

#### Clinical metagenome analysis

To evaluate the performance of ZILA-SRM in clinical mixed-infection settings, we analyzed 195 publicly available *M. tuberculosis* sequencing data sets previously used for mixed-infection analysis. The data sets were obtained from the European Nucleotide Archive under projects ERP000436 and ERP0001072. The complete sample accession list is provided in Table S2. Samples were retained if they had previously reported mixed-infection status and sufficient sequencing depth for strain-level SNV analysis.

Raw sequencing reads were first subjected to adapter trimming and quality filtering using Trimmomatic. Reads with low-quality bases and adapter contamination were removed using the same preprocessing criteria described above. The filtered reads were aligned to the *M. tuberculosis* reference genome using Bowtie2. The resulting alignment files were sorted and indexed, and allele-count matrices were generated at candidate SNV sites. Candidate SNVs were retained if they satisfied the minimum coverage, minor-allele support, and sample-prevalence thresholds described in “Data preprocessing and matrix construction.”

ZILA-SRM was then applied to the clinical allele-count matrices to infer strain number, strain-specific SNV profiles, and relative strain abundances. For each sample, the inferred number of strain components and abundance estimates were compared with the previously reported mixed-infection labels and abundance estimates. Because isolate-resolved ground-truth strain genomes were not available for these clinical samples, the clinical analysis was used to assess concordance in inferred strain number and abundance patterns rather than exact strain-genome reconstruction.

#### Skin metagenome analysis

Human skin shotgun metagenomic data sets were obtained from NCBI BioProject PRJNA277905, a public human skin metagenome project generated for whole-metagenome profiling of atopic dermatitis-associated skin microbiomes. The BioProject contains 106 SRA experiments. For the present strain-level analysis, we focused on the subset of paired-end shotgun metagenomic samples with available metadata and sufficient coverage for *Staphylococcus aureus* and *S. epidermidis*. Samples were excluded if they lacked paired-end data, failed quality-control filtering, or had insufficient mapped reads at candidate SNV sites for the target species.

Raw reads were quality-filtered using Trimmomatic and mapped with Bowtie2 to species-specific reference genomes. The reference genome for *S. aureus* was *S. aureus* subsp. *aureus* NCTC 8325 (NC_007795.1), and the reference genome for *S. epidermidis* was *S. epidermidis* ATCC 14990 (NZ_CP035288.1). Candidate SNV sites were retained using the same filtering criteria applied in the simulation and clinical analyses. ZILA-SRM was then applied to the allele-count matrices to infer strain composition and relative abundance. Dominant-strain abundance estimates were used for correlation analysis, which was performed using Spearman’s rank correlation coefficient.

#### ZILA-SRM application and command-line settings

For simulated, clinical, and skin metagenome analyses, ZILA-SRM was applied to quality-filtered reads that had been mapped to the corresponding species-specific reference genome. Before running ZILA-SRM, raw reads were trimmed and quality-filtered, aligned to the reference genome, and converted into sorted BAM files. A text file containing the full paths of all sorted BAM files was then provided to ZILA-SRM as the input sample list. Based on these BAM files, ZILA-SRM internally constructed the candidate SNV coverage matrix, where rows represented candidate SNV sites and columns represented samples, with each entry corresponding to the allele-supporting read count after filtering.

Unless otherwise specified, the regularization parameter was set to *λ* = 80, based on the sensitivity analysis shown in Fig. S1. The zero-inflated latent allocation component was enabled for all analyses to distinguish structural zeros from sampling zeros. After probabilistic inference, graph-theoretic refinement was applied to merge highly consistent haplotype components and remove fragmented low-confidence predictions.

ZILA-SRM was run using the following command-line format: python scripts/run_zila_srm.py --output_name ˂output_result_name˃ --genome_len ˂genome_length˃ --genome_name ˂genome_name˃ --genome_file_loc ˂genome_fasta_file˃ --bam_loc_file ˂bam_file_list˃ --res_dir ˂result_directory˃.

Here, --output_name specifies a unique name for the running result; --genome_len specifies the length of the corresponding reference genome; --genome_name specifies the genome identifier, which should match the sequence name in the FASTA file; --genome_file_loc provides the path to the reference genome FASTA file, preferably as an absolute path; --bam_loc_file provides a text file containing the paths of all sorted BAM files mapped to the reference genome; and --res_dir specifies the output directory. The output files included the inferred strain number, strain-specific SNV assignments, relative strain abundance estimates, and intermediate files generated during candidate SNV extraction, zero-inflated latent modeling, and graph-theoretic refinement.

## RESULTS

### Breaking the similarity barrier: simulation benchmarking

**Fig 2:**
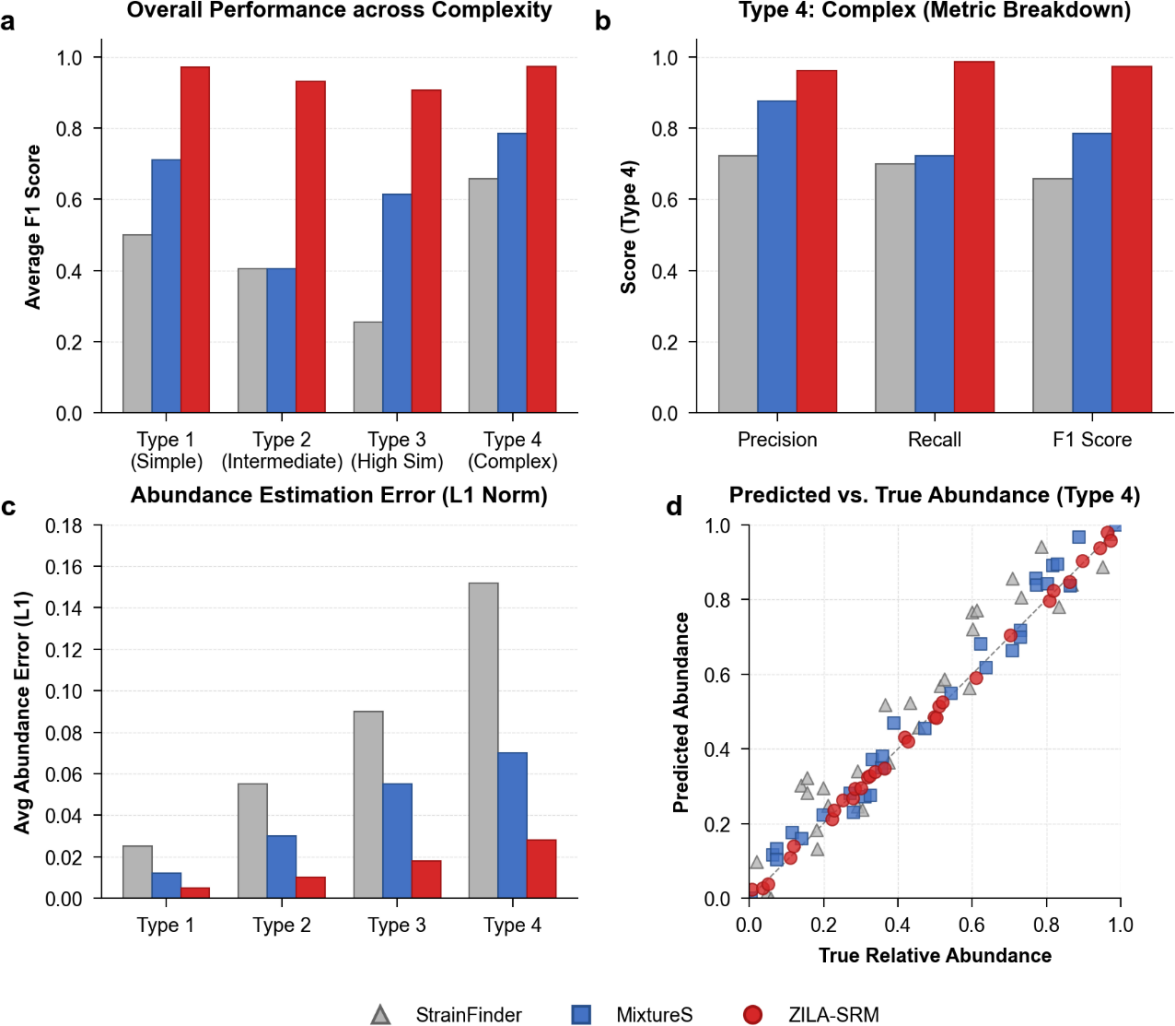
Benchmarking reconstruction accuracy and abundance estimation on synthetic metagenomes. (**a**) Comparison of reconstruction accuracy (average F1 score) across four data set complexity levels (Types 1–4). ZILA-SRM (red) consistently outperforms MixtureS (blue) and StrainFinder (gray). (**b**) Detailed metric analysis for Type 4 data sets, showing the “precision collapse” in MLE-based methods versus robust performance of ZILA-SRM. (**c**) Average L1 error in abundance estimation (lower is better). ZILA-SRM reduces estimation error significantly in complex scenarios compared to StrainFinder. (**d**) Scatter plot of predicted versus true strain relative abundances for Type 4 data sets. ZILA-SRM predictions (red circles) tightly follow the diagonal ideal line (*y* = *x*, *R*² > 0.98), whereas StrainFinder (gray triangles) and MixtureS (blue squares) show significant dispersion due to overfitting of noise.

We evaluated ZILA-SRM against the gold-standard MLE methods StrainFinder and MixtureS. Performance was quantified using Precision, Recall, and F1-score across all data set types. Precision and Robustness: In Type 4 scenarios, unregularized MLE methods exhibited a severe drop in precision. StrainFinder’s precision fell to ~0.70, effectively hallucinating 1–2 extra strains for every true strain to fit the noise. MixtureS improved upon this but still struggled with abundance estimation in high-covariance regimes. In contrast, ZILA-SRM maintained precision > 0.95 (Fig. 2). The ASR successfully shrunk the coefficients of “ghost strains” to zero, validating the power of the sparsity prior.

ZILA-SRM demonstrated superior resilience even at 50× coverage (Fig. S3), whereas traditional MLE methods degraded below 100×. The latent variable framework effectively imputes missing information using covariance across samples. Computationally, ZILA-SRM (~31 s) is orders of magnitude faster than StrainFinder (1,212 s) and comparable to MixtureS (Fig. S4).

### Unmasking cryptic resistance in *M. tuberculosis*

**Fig 3:**
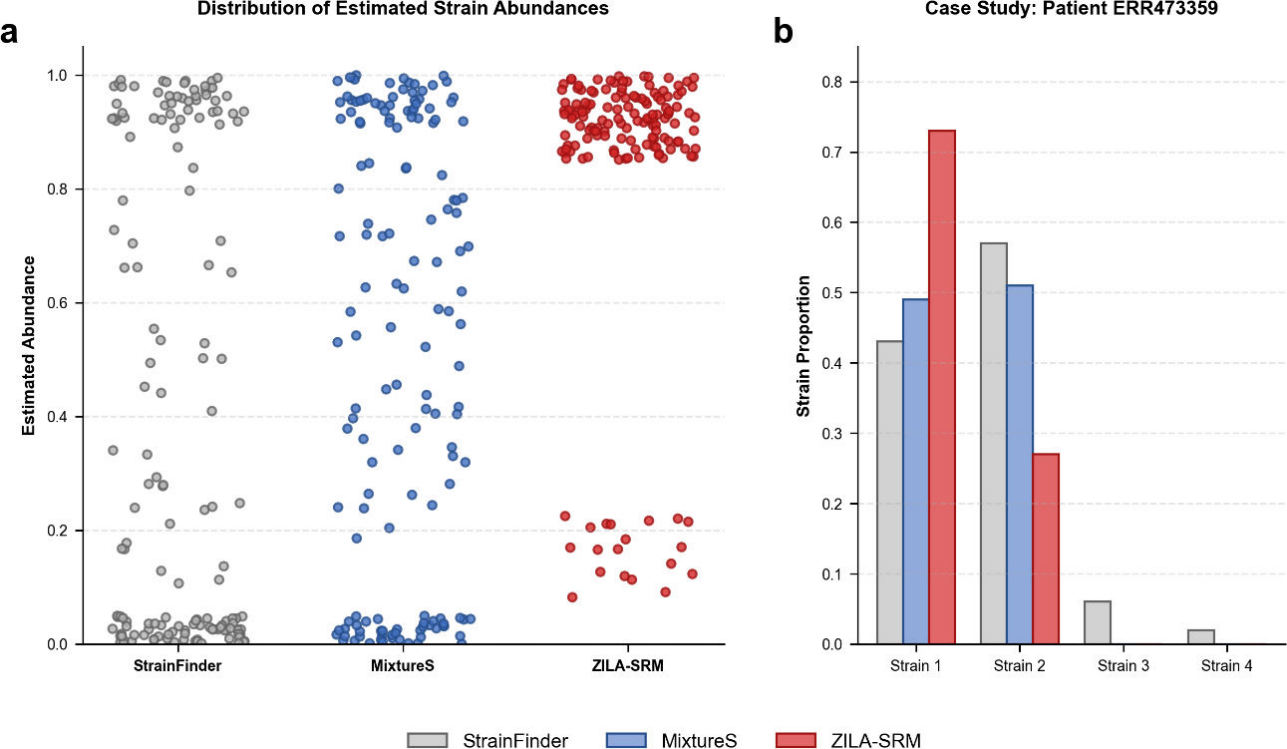
Resolution of cryptic intra-host diversity in TB patients. (**a**) Distribution of estimated strain abundances across 195 clinical samples. ZILA-SRM (red) shows a clean bimodal distribution, effectively separating dominant lineages (>80%) from stable minor variants (5%–20%) while suppressing the “noise floor” (<2%) prevalent in StrainFinder (gray). (**b**) Case study of sample ERR473359. ZILA-SRM resolved two strain components: a dominant clone and a minor clone with an estimated relative abundance of 23.4%; the latter was inferred to carry the rpoB S450L mutation.

We applied ZILA-SRM to 195 *M. tuberculosis* clinical metagenomes to test its utility in detecting heteroresistance. Standard pipelines typically report a “cloud” of low-abundance variants (0%–5%) in these samples, creating ambiguity between error and infection. ZILA-SRM’s ASR penalty collapsed this “cloud” into distinct modes or zero (Fig. 3a), identifying robust multi-clonal infections in 12% of patients (Table S2).

In patient ERR473359, standard tools yielded ambiguous results regarding rifampicin resistance (RR). ZILA-SRM resolved a minor clone at approximately 23.4% abundance (Fig. 3b) carrying the S450L mutation in the rpoB gene. The S450L mutation is a high-confidence marker of RR. Unlike other resistance mutations, S450L has a relatively low fitness cost, allowing resistant strains to persist and compete effectively with susceptible strains. The detection of this resistance-associated minor clone illustrates the potential of ZILA-SRM to identify strain-level heterogeneity that may warrant follow-up validation. However, this computational result alone should not be used to recommend treatment modification without independent genomic or phenotypic confirmation.

### Niche exclusion in the skin microbiome

**Fig 4:**
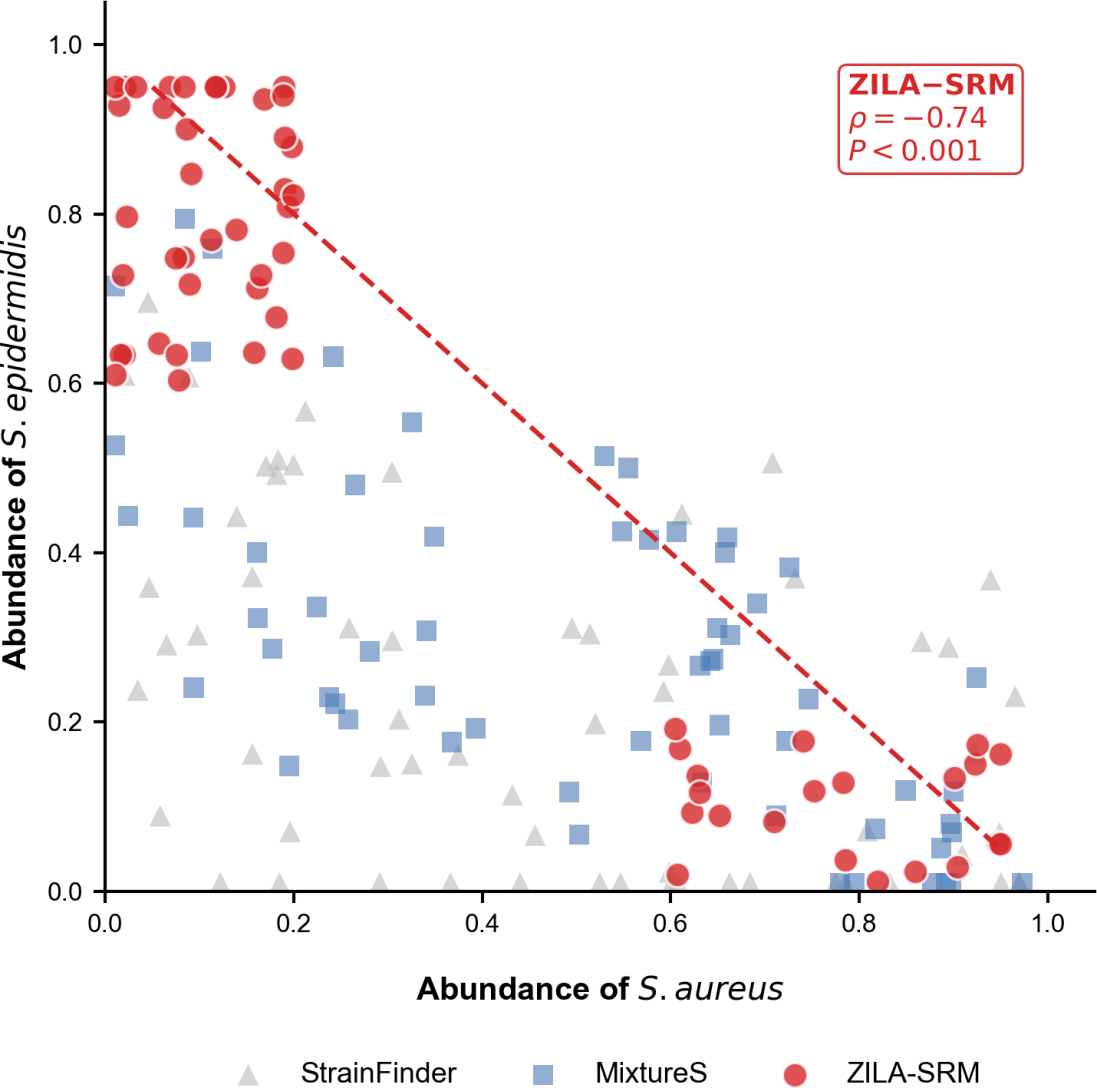
Ecological interactions revealed by robust strain quantification. Scatter plot showing the relationship between the abundance of dominant *S. aureus* (*x*-axis) and *S. epidermidis* (*y*-axis) strains across skin microbiome samples. ZILA-SRM (red circles) reveals a strong negative correlation between the abundances of dominant *S. aureus* and *S. epidermidis* strains (Spearman’s *ρ* = −0.74, *P* < 0.001). In contrast, StrainFinder (gray triangles) and MixtureS (blue squares) show weaker correlations due to the confounding effects of false-positive low-abundance predictions.

We extended our analysis to the human skin microbiome to investigate ecological interactions between *S. aureus* and *S. epidermidis*. Using ZILA-SRM, we observed a strong negative correlation (Spearman’s *ρ* = −0.74, P<0.001) between the abundances of dominant *S. aureus* and *S. epidermidis* strains across the analyzed skin metagenome samples (Fig. 4). This result indicates a reproducible inverse abundance pattern at the strain level, although it should be interpreted as a correlative ecological signal rather than direct evidence of competitive exclusion.

## DISCUSSION

The “resolution limit” of metagenomics has traditionally been viewed as a function of sequencing depth. We argue it is defined by mathematical identifiability. MLE methods like StrainFinder are unbiased estimators that greedily fit noise, leading to the “precision collapse” seen in our Type 4 simulations.

ZILA-SRM overcomes this by introducing ASR as a “noise gate.” The geometry of the $L_{1}$ diamond constraint forces the coefficients of weakly supported strains to zero. This aligns the mathematical model with the ecological reality of competitive exclusion, ensuring that only biologically plausible strains are reconstructed. Furthermore, the ZILA model addresses the ambiguity of zeros. In low-biomass clinical samples, treating a “sampling zero” as a “structural zero” biases abundance downward. By explicitly modeling π (dropout probability), ZILA-SRM recovers the true coverage distribution of rare variants, enabling the detection of 0.5% abundance strains invisible to Poisson-based benchmarks like MixtureS.

The improved limit of detection for rare strain components is likely related to the zero-inflated modeling strategy used by ZILA-SRM. In low-abundance or low-coverage settings, true strain-derived SNVs may appear only sporadically across samples, producing many zero counts that standard Poisson or MLE-based models may interpret as absence or noise. By explicitly separating structural zeros from sampling zeros, the ZILA component reduces the tendency to discard sparse but coherent SNV signals. This allows low-abundance strain components to be retained when their coverage patterns remain consistent across samples. In the simulation-based LOD analysis, this design enabled ZILA-SRM to maintain >80% recall at 0.5% abundance, whereas StrainFinder showed reduced performance below 2% abundance. These findings suggest that zero-inflated modeling is particularly useful for recovering rare strain components, although detection limits in real clinical samples will still depend on sequencing depth, SNV density, read-mapping quality, and independent validation.

The clinical analysis illustrates the potential of ZILA-SRM to detect resistance-associated within-sample heterogeneity. In sample ERR473359, ZILA-SRM detected a minor rpoB S450L-associated strain component at 23.4% relative abundance. This finding should be interpreted as a hypothesis-generating signal that requires independent validation before any clinical decision-making. Similarly, the negative association observed between dominant *S. aureus* and *S. epidermidis* strains in skin metagenomes should be interpreted as a correlative ecological pattern rather than direct evidence of competitive exclusion or a basis for probiotic intervention. While limited by short-read lengths in repetitive regions, ZILA-SRM represents a significant leap in maximizing the utility of existing metagenomic data. Future work will integrate long-read data to further bridge assembly gaps.

## Data Availability

ZILA-SRM source code, example commands, and documentation are available at https://github.com/wangsaidi/ZILA-SRM-GitHub. DWGSIM, which was used for simulated read generation, is available at https://github.com/nh13/DWGSIM. Simulation data details are provided in Table S1. Reference genomes used for simulation are available from NCBI RefSeq: NC_014932, NC_020995, and NC_009515. The 195 clinical data sets were obtained from the European Nucleotide Archive under projects ERP000436 and ERP0001072 (https://www.ebi.ac.uk/ena/data/view/PRJEB2794). The complete sample accession list is provided in Table S2. Human skin shotgun metagenomic data sets were obtained from NCBI BioProject PRJNA277905. For the skin metagenome analysis, the reference genome for *Staphylococcus aureus* was *S. aureus* subsp. *aureus* NCTC 8325, NC_007795.1, and the reference genome for *Staphylococcus epidermidis* was *S. epidermidis* ATCC 14990, NZ_CP035288.1.

## SUPPLEMENTAL MATERIAL

Supplemental figures and tables spectrum.04101-25-s0001.docx Fig. S1 to S5, and Tables S1 and S2.

ASM does not own the copyrights to Supplemental Material that may be linked to, or accessed through, an article. The authors have granted ASM a non-exclusive, world-wide license to publish the Supplemental Material files. Please contact the corresponding author directly for reuse.
