# Supplementary material for: ZILA-SRM: a probabilistic framework with zero-inflated latent models for robust strain reconstruction from metagenomes
Source: Microbiol Spectr. 2026 Jun 30;14(8):e04101-25. doi: 10.1128/spectrum.04101-25 (PMC13436319; doi:10.1128/spectrum.04101-25)
Supplement: Supplementary file 1 [file spectrum.04101-25-s0001.docx]

****Supplementary Figure 1 | Sensitivity analysis of the regularization hyperparameter λ.**** The F1-score of strain reconstruction was evaluated on Type 3 (Blue dashed) and Type 4 (Red solid) simulated datasets across a range of λ values. The performance follows a convex trajectory: low λ values (<30) lead to overfitting (high False Positives), while excessive regularization (λ > 150) causes underfitting (loss of True Positives). The optimal stability λ is observed around 80, which was selected as the default for all subsequent analyses.

****Supplementary Figure 2 | Limit of Detection (LOD) analysis for rare variants.**** Recall rates were measured for simulated strains with varying relative abundances (0.1% to 5%). **ZILA**-SRM**** (Red) maintains a recall of >80% even at 0.5% abundance, significantly outperforming ****StrainFinder**** (Grey), which requires >2% abundance for reliable detection. This enhanced sensitivity is attributable to the ****ZILA**** model's ability to distinguish low-coverage signal from stochastic dropout.

****Supplementary Figure 3 | Robustness to sequencing depth variation.**** Comparison of reconstruction accuracy (F1 Score) at different average sequencing depths (50X–300X). ****ZILA-SRM**** demonstrates superior resilience at lower coverage (50X), where the Zero-Inflated Poisson model effectively compensates for data sparsity. Traditional MLE methods show marked performance degradation when depth falls below 100X.

****Supplementary Figure 4 | Computational efficiency benchmarking. a****, Average runtime (in seconds) on a standard workstation (16 cores) for processing a dataset with ~1M reads. ****SMS**** (Red) takes slightly longer (31s) than ****MixtureS**** (28s) due to the two-step process but is orders of magnitude faster than ****StrainFinder**** (1212s). ****b****, Peak memory usage (GB). **ZILA-SRM** utilizes sparse matrix operations, resulting in a computational footprint comparable to MixtureS and significantly more efficient than StrainFinder.

****Supplementary Figure 5 | Decision Boundary of the Hybrid Discriminant Kernel.**** Visualization of the learned metric kernel for strain differentiation. The scatter plot displays the distribution of SNV pairs in the feature space defined by ****Euclidean Distance (d)**** and ****Kendall Rank Correlation (c)****. ****Blue points**** represent pairs originating from the same strain (characterized by low distance and high correlation), while ****Red points**** represent pairs from different strains. The contours and the solid black line indicate the decision boundary (K(u, v) = 0) learned via supervised metric learning on the training corpus. The non-linear boundary effectively separates the two classes, validating the discriminative power of the hybrid kernel formula.

Table S1. Simulated datasets.

| Types | Strain Configuration | #strains | coverage |
| --- | --- | --- | --- |
| Type1 | 10:20:30:40 | 4 | 100x |
|  | 10:30:60 | 3 | 100x |
|  | 15:30:55 | 3 | 100x |
|  | 30:70 | 2 | 100x |
|  | 10:25:25:40 | 4 | 100x |
|  | 10:25:25:40 | 4 | 150x |
|  | 10:25:25:40 | 4 | 200x |
|  | 10:25:25:40 | 4 | 300x |
| Type2 | 10:20:30:40 | 4 | 100x |
|  | 10:25:25:40 | 4 | 100x |
|  | 10:25:25:40 | 4 | 150x |
|  | 10:25:25:40 | 4 | 200x |
|  | 10:25:25:40 | 4 | 300x |
| Type3 | 10:20:30:40 | 4 | 100x |
|  | 10:25:25:40 | 4 | 100x |
|  | 10:25:25:40 | 4 | 150x |
|  | 10:25:25:40 | 4 | 200x |
|  | 10:25:25:40 | 4 | 300x |
| Type4 | 10:30:60 | 3 | 100x |
|  | 15:30:55 | 3 | 100x |
|  | 10:30:60 | 3 | 150x |
|  | 10:30:60 | 3 | 200x |
|  | 10:30:60 | 3 | 300x |
|  | 15:30:55 | 3 | 150x |
|  | 15:30:55 | 3 | 200x |
|  | 15:30:55 | 3 | 300x |

There are 27 datasets generated in each row, each of which corresponds to one of the three different bacterial species (species 1: *Bartonella clarridgeiae* NC_014932, species 2: *Enterococcus casseli flavus* NC_020995, and species 3: *Methanobrevibacter smithii* NC_009515) and one of the nine different sample numbers (5, 8, 10, 12, 15, 20, 25, 30, 35). The number and order of strains in each dataset are specified by the strain configuration. For instance, the configuration 10:20:30:40 in the first row specifies that there are four strains, with the coverage of the 1^st^, 2^nd^, 3^rd^ and 4^th^ strain as 10X, 20X, 30X and 40X,respectively. There is no shared SNPs among strains in Type 1 datasets. In each type 2 dataset, the first two strains share 30% of their SNPs, the first three strains share 20% of their SNPs, and the fourth strain shares no SNP with other strains. In each type 3 dataset, the first two strains share 30% of their SNPs, the first three strains share 10% of their SNPs, and there is no SNP shared between the first three strains and the last strain. In each type 4 dataset, the first two strains share 30% of their SNPs, and the third strain share no SNP with other strains.

Table S2. 195 TB abundance result from three tools.

| sample | # of verified method in refernce paper | major strain proportion | MixtureS | ZILA-SRM | StrainFinder |
| --- | --- | --- | --- | --- | --- |
| ERR036194 | single | 1 | 2(0.349) | 2(0.071) | 2(0.459) |
| ERR036233 | both | 0.72 | 4(0.113) | 2(0.124) | 2(0.215) |
| ERR036248 | both | 0.88 | 2(0.01) | 2(0.045) | 2(0.114) |
| ERR037469 | both | 0.63 | 2(0.144) | 2(0.001) | 2(0.115) |
| ERR037547 | both | 0.85 | 2(0.042) | 2(0.025) | 2(0.035) |
| ERR126641 | both | 0.84 | 4(0.137) | 2(0.025) | 2(0.063) |
| ERR126642 | both | 0.8 | 2(0.088) | 2(0.161) | 2(0.055) |
| ERR161024 | both | 0.86 | 3(0.082) | 2(0.033) | 2(0.003) |
| ERR161026 | both | 0.85 | 3(0.106) | 2(0.182) | 2(0.066) |
| ERR161027 | both | 0.82 | 3(0.06) | 2(0.081) | 2(0.046) |
| ERR161034 | both | 0.65 | 3(0.086) | 2(0.332) | 2(0.067) |
| ERR161039 | both | 0.63 | 4(0.157) | 2(0.352) | 2(0.084) |
| ERR161049 | both | 0.87 | 3(0.099) | 2(0.209) | 2(0.246) |
| ERR161050 | both | 0.73 | 5(0.218) | 2(0.214) | 2(0.214) |
| ERR161055 | both | 0.89 | 2(0.005) | 2(0.022) | 2(0.387) |
| ERR161071 | both | 0.84 | 4(0.148) | 2(0.318) | 2(0.008) |
| ERR161077 | both | 0.78 | 5(0.238) | 2(0.116) | 2(0.206) |
| ERR161078 | both | 0.58 | 4(0.123) | 2(0.054) | 2(0.038) |
| ERR161081 | both | 0.88 | 2(0.124) | 2(0.105) | 2(0.317) |
| ERR161084 | both | 0.87 | 3(0.103) | 2(0.349) | 2(0.047) |
| ERR161088 | both | 0.88 | 3(0.11) | 2(0.175) | 1(0.12) |
| ERR161090 | both | 0.91 | 2(0.039) | 2(0.062) | 2(0.406) |
| ERR161091 | both | 0.89 | 2(0.003) | 2(0.08) | 2(0.336) |
| ERR161097 | both | 0.85 | 2(0.025) | 2(0.131) | 2(0.057) |
| ERR161120 | both | 0.86 | 2(0.104) | 2(0.128) | 2(0.177) |
| ERR161122 | both | 0.87 | 3(0.099) | 2(0.187) | 2(0.239) |
| ERR161123 | both | 0.81 | 3(0.056) | 2(0.166) | 2(0.042) |
| ERR161170 | both | 0.89 | 2(0.228) | 2(0.094) | 2(0.352) |
| ERR161173 | both | 0.9 | 2(0.265) | 2(0.086) | 2(0.378) |
| ERR161176 | both | 0.88 | 3(0.1) | 2(0.101) | 2(0.375) |
| ERR161184 | both | 0.87 | 3(0.196) | 2(0.114) | 2(0.349) |
| ERR161194 | both | 0.86 | 3(0.101) | 2(0.07) | 2(0.121) |
| ERR161195 | both | 0.88 | 2(0.01) | 2(0.104) | 2(0.377) |
| ERR163932 | both | 0.91 | 3(0.097) | 2(0.201) | 2(0.401) |
| ERR163940 | both | 0.87 | 2(0.04) | 2(0.109) | 2(0.364) |
| ERR163942 | both | 0.87 | 3(0.094) | 2(0.078) | 2(0.144) |
| ERR163943 | both | 0.83 | 3(0.087) | 2(0.011) | 2(0.101) |
| ERR163947 | both | 0.5 | 3(0.122) | 2(0.211) | 2(0.04) |
| ERR163954 | both | 0.92 | 2(0.155) | 2(0.208) | 2(0.417) |
| ERR163971 | both | 0.89 | 2(0.007) | 2(0.021) | 2(0.388) |
| ERR163986 | both | 0.9 | 2(0.018) | 2(0.398) | 2(0.394) |
| ERR163996 | both | 0.88 | 3(0.098) | 2(0.042) | 2(0.377) |
| ERR164007 | both | 0.88 | 2(0.01) | 2(0.334) | 2(0.167) |
| ERR164021 | both | 0.7 | 3(0.043) | 2(0.001) | 2(0.187) |
| ERR176446 | both | 0.88 | 4(0.152) | 2(0.1) | 2(0.379) |
| ERR176458 | both | 0.82 | 4(0.16) | 2(0.29) | 2(0.316) |
| ERR176460 | both | 0.88 | 2(0.001) | 2(0.27) | 2(0.373) |
| ERR176461 | both | 0.8 | 2(0.06) | 2(0.189) | 2(0.298) |
| ERR176514 | single | 1 | 2(0.282) | 2(0.048) | 2(0.48) |
| ERR176521 | both | 0.89 | 2(0.132) | 2(0.182) | 2(0.385) |
| ERR176533 | both | 0.9 | 2(0.001) | 2(0.036) | 2(0.275) |
| ERR176549 | both | 0.72 | 2(0.053) | 2(0.071) | 2(0.213) |
| ERR176556 | both | 0.86 | 2(0.041) | 2(0.032) | 2(0.113) |
| ERR176557 | both | 0.86 | 2(0.058) | 2(0.081) | 2(0.013) |
| ERR176600 | both | 0.89 | 2(0.014) | 2(0.096) | 2(0.386) |
| ERR176604 | both | 0.89 | 3(0.111) | 2(0.384) | 2(0.369) |
| ERR176610 | both | 0.9 | 2(0.026) | 2(0.089) | 2(0.397) |
| ERR176611 | both | 0.88 | 3(0.1) | 2(0.106) | 2(0.376) |
| ERR176616 | both | 0.63 | 5(0.191) | 2(0.333) | 2(0.125) |
| ERR176620 | both | 0.54 | 3(0.096) | 2(0.378) | 2(0.015) |
| ERR176621 | both | 0.9 | 2(0.002) | 2(0.039) | 2(0.273) |
| ERR176631 | both | 0.89 | 3(0.099) | 2(0.089) | 2(0.388) |
| ERR176650 | both | 0.87 | 4(0.152) | 2(0.225) | 2(0.363) |
| ERR176652 | both | 0.82 | 5(0.245) | 2(0.097) | 2(0.304) |
| ERR176653 | both | 0.8 | 4(0.178) | 2(0.154) | 2(0.29) |
| ERR176655 | both | 0.91 | 2(0.208) | 2(0.251) | 2(0.403) |
| ERR176664 | both | 0.88 | 3(0.229) | 2(0.166) | 2(0.378) |
| ERR176668 | both | 0.92 | 2(0.276) | 2(0.038) | 2(0.395) |
| ERR176672 | both | 0.89 | 2(0.237) | 2(0.201) | 2(0.371) |
| ERR176681 | both | 0.89 | 2(0.187) | 2(0.068) | 2(0.388) |
| ERR176688 | both | 0.9 | 2(0.253) | 2(0.077) | 2(0.381) |
| ERR176701 | both | 0.83 | 4(0.165) | 2(0.159) | 2(0.327) |
| ERR176703 | single | 1 | 2(0.348) | 2(0.494) | 2(0.493) |
| ERR176706 | both | 0.89 | 2(0.178) | 2(0.073) | 2(0.362) |
| ERR176709 | both | 0.65 | 5(0.171) | 2(0.042) | 2(0.142) |
| ERR176713 | both | 0.89 | 4(0.194) | 2(0.265) | 2(0.345) |
| ERR176723 | both | 0.88 | 3(0.103) | 2(0.043) | 2(0.361) |
| ERR176725 | both | 0.88 | 4(0.152) | 2(0.181) | 2(0.37) |
| ERR176734 | both | 0.91 | 2(0.017) | 2(0.063) | 2(0.375) |
| ERR176738 | both | 0.88 | 4(0.193) | 2(0.184) | 2(0.379) |
| ERR176746 | both | 0.87 | 2(0.176) | 2(0.053) | 2(0.359) |
| ERR176748 | both | 0.87 | 2(0.031) | 2(0.119) | 2(0.09) |
| ERR176749 | both | 0.81 | 2(0.035) | 2(0.175) | 2(0.023) |
| ERR176755 | both | 0.89 | 2(0.179) | 2(0.055) | 2(0.375) |
| ERR176785 | single | 1 | 2(0.32) | 2(0.172) | 2(0.479) |
| ERR176793 | both | 0.54 | 5(0.198) | 2(0.146) | 2(0.004) |
| ERR176796 | both | 0.88 | 2(0.118) | 2(0.072) | 2(0.374) |
| ERR176802 | both | 0.89 | 2(0.217) | 2(0.187) | 2(0.347) |
| ERR176807 | both | 0.87 | 3(0.098) | 2(0.164) | 2(0.367) |
| ERR176809 | both | 0.89 | 2(0.202) | 2(0.064) | 2(0.387) |
| ERR176810 | both | 0.91 | 2(0.214) | 2(0.072) | 2(0.409) |
| ERR176813 | both | 0.88 | 3(0.106) | 2(0.364) | 2(0.377) |
| ERR181686 | both | 0.87 | 3(0.094) | 2(0.183) | 2(0.079) |
| ERR181688 | both | 0.83 | 2(0.041) | 2(0.153) | 2(0.249) |
| ERR181689 | both | 0.85 | 2(0.044) | 2(0.148) | 2(0.069) |
| ERR181695 | both | 0.87 | 2(0.013) | 2(0.277) | 2(0.368) |
| ERR181705 | both | 0.84 | 2(0.044) | 2(0.231) | 2(0.028) |
| ERR181708 | both | 0.87 | 3(0.092) | 2(0.027) | 2(0.321) |
| ERR181749 | both | 0.87 | 2(0.209) | 2(0.352) | 2(0.285) |
| ERR181750 | both | 0.89 | 2(0.078) | 2(0.045) | 2(0.359) |
| ERR181752 | both | 0.85 | 2(0.007) | 2(0.116) | 2(0.317) |
| ERR181753 | both | 0.87 | 2(0.004) | 2(0.363) | 1(0.13) |
| ERR181782 | both | 0.86 | 3(0.085) | 2(0.114) | 2(0.042) |
| ERR181784 | both | 0.82 | 3(0.055) | 2(0.131) | 2(0.047) |
| ERR181785 | both | 0.8 | 2(0.086) | 2(0.267) | 2(0.027) |
| ERR181810 | both | 0.9 | 2(0.23) | 2(0.007) | 2(0.375) |
| ERR181811 | both | 0.54 | 5(0.191) | 2(0.413) | 2(0.0) |
| ERR181813 | both | 0.62 | 4(0.121) | 2(0.072) | 2(0.084) |
| ERR181827 | both | 0.88 | 3(0.096) | 2(0.2) | 2(0.374) |
| ERR181828 | both | 0.88 | 3(0.092) | 2(0.194) | 2(0.319) |
| ERR181838 | both | 0.88 | 2(0.004) | 2(0.108) | 2(0.378) |
| ERR181845 | both | 0.9 | 2(0.035) | 2(0.051) | 2(0.397) |
| ERR181849 | both | 0.88 | 3(0.095) | 2(0.111) | 2(0.361) |
| ERR181866 | both | 0.89 | 3(0.104) | 2(0.101) | 1(0.11) |
| ERR181870 | both | 0.9 | 2(0.024) | 2(0.085) | 2(0.398) |
| ERR181876 | both | 0.91 | 2(0.1913) | 2(0.078) | 2(0.2058) |
| ERR181878 | both | 0.81 | 2(0.101) | 2(0.178) | 2(0.293) |
| ERR181880 | both | 0.86 | 2(0.011) | 2(0.124) | 2(0.341) |
| ERR181881 | both | 0.84 | 4(0.146) | 2(0.292) | 2(0.129) |
| ERR181909 | both | 0.89 | 2(0.037) | 2(0.092) | 2(0.378) |
| ERR181913 | both | 0.89 | 2(0.179) | 2(0.097) | 2(0.362) |
| ERR181923 | both | 0.88 | 2(0.015) | 2(0.073) | 1(0.12) |
| ERR181933 | both | 0.91 | 2(0.043) | 2(0.003) | 2(0.409) |
| ERR181937 | both | 0.87 | 2(0.012) | 2(0.059) | 2(0.349) |
| ERR181945 | both | 0.87 | 4(0.145) | 2(0.067) | 2(0.364) |
| ERR181953 | both | 0.87 | 2(0.013) | 2(0.092) | 2(0.368) |
| ERR181974 | both | 0.85 | 3(0.094) | 2(0.146) | 2(0.219) |
| ERR181977 | both | 0.8 | 4(0.143) | 2(0.096) | 2(0.056) |
| ERR181983 | both | 0.9 | 3(0.094) | 2(0.399) | 2(0.373) |
| ERR182015 | both | 0.85 | 4(0.143) | 2(0.342) | 2(0.176) |
| ERR182026 | both | 0.84 | 4(0.151) | 2(0.01) | 2(0.064) |
| ERR182027 | both | 0.87 | 4(0.138) | 2(0.208) | 2(0.167) |
| ERR182041 | both | 0.88 | 2(0.011) | 2(0.065) | 2(0.368) |
| ERR182049 | both | 0.89 | 3(0.098) | 2(0.091) | 2(0.366) |
| ERR190340 | both | 0.63 | 5(0.225) | 2(0.356) | 2(0.127) |
| ERR190342 | both | 0.86 | 3(0.09) | 2(0.185) | 2(0.165) |
| ERR190343 | both | 0.8 | 3(0.045) | 2(0.215) | 2(0.029) |
| ERR190379 | both | 0.77 | 4(0.111) | 2(0.239) | 2(0.2) |
| ERR190388 | both | 0.91 | 3(0.268) | 2(0.319) | 1(0.09) |
| ERR211990 | both | 0.89 | 3(0.101) | 2(0.084) | 2(0.343) |
| ERR212002 | both | 0.86 | 3(0.106) | 2(0.065) | 2(0.323) |
| ERR212004 | both | 0.86 | 4(0.157) | 2(0.331) | 2(0.336) |
| ERR212041 | both | 0.85 | 2(0.162) | 2(0.165) | 2(0.337) |
| ERR212058 | both | 0.88 | 3(0.11) | 2(0.09) | 2(0.349) |
| ERR212059 | both | 0.86 | 3(0.11) | 2(0.356) | 2(0.356) |
| ERR212069 | both | 0.86 | 3(0.092) | 2(0.0) | 2(0.312) |
| ERR212086 | both | 0.88 | 2(0.021) | 2(0.179) | 2(0.129) |
| ERR212098 | both | 0.84 | 3(0.073) | 2(0.109) | 2(0.066) |
| ERR212100 | both | 0.84 | 3(0.073) | 2(0.309) | 2(0.027) |
| ERR212101 | both | 0.85 | 3(0.076) | 2(0.174) | 2(0.009) |
| ERR212107 | both | 0.87 | 3(0.092) | 2(0.112) | 2(0.211) |
| ERR212112 | both | 0.89 | 4(0.191) | 2(0.184) | 2(0.357) |
| ERR212134 | both | 0.85 | 2(0.007) | 2(0.143) | 2(0.328) |
| ERR212161 | both | 0.87 | 3(0.102) | 2(0.356) | 2(0.336) |
| ERR212165 | both | 0.85 | 3(0.09) | 2(0.01) | 2(0.266) |
| ERR216899 | both | 0.88 | 4(0.212) | 2(0.033) | 2(0.367) |
| ERR216913 | both | 0.88 | 2(0.004) | 2(0.37) | 2(0.371) |
| ERR216914 | both | 0.75 | 5(0.235) | 2(0.236) | 2(0.225) |
| ERR216917 | both | 0.89 | 4(0.201) | 2(0.083) | 2(0.381) |
| ERR216932 | both | 0.89 | 3(0.233) | 2(0.084) | 2(0.387) |
| ERR216933 | both | 0.9 | 2(0.243) | 2(0.084) | 2(0.341) |
| ERR216942 | both | 0.87 | 2(0.019) | 2(0.353) | 2(0.366) |
| ERR216952 | both | 0.91 | 4(0.228) | 2(0.007) | 1(0.09) |
| ERR216956 | both | 0.89 | 3(0.153) | 2(0.197) | 2(0.386) |
| ERR216961 | both | 0.93 | 3(0.217) | 2(0.051) | 2(0.392) |
| ERR216966 | both | 0.88 | 3(0.131) | 2(0.006) | 2(0.316) |
| ERR216967 | both | 0.88 | 3(0.13) | 2(0.215) | 2(0.376) |
| ERR216971 | both | 0.69 | 6(0.211) | 2(0.033) | 2(0.187) |
| ERR216974 | both | 0.89 | 3(0.145) | 2(0.186) | 2(0.223) |
| ERR216977 | both | 0.89 | 3(0.167) | 2(0.021) | 2(0.387) |
| ERR216983 | both | 0.89 | 3(0.139) | 2(0.322) | 2(0.386) |
| ERR216984 | both | 0.88 | 3(0.123) | 2(0.218) | 2(0.374) |
| ERR216989 | both | 0.87 | 3(0.151) | 2(0.099) | 2(0.238) |
| ERR221524 | both | 0.88 | 2(0.005) | 2(0.097) | 2(0.336) |
| ERR221534 | single | 1 | 2(0.102) | 2(0.47) | 2(0.465) |
| ERR221536 | both | 0.87 | 3(0.101) | 2(0.081) | 2(0.249) |
| ERR221538 | both | 0.88 | 4(0.16) | 2(0.351) | 2(0.368) |
| ERR221539 | both | 0.82 | 3(0.073) | 2(0.284) | 2(0.105) |
| ERR221561 | both | 0.69 | 4(0.115) | 2(0.132) | 2(0.189) |
| ERR221567 | both | 0.87 | 3(0.107) | 2(0.369) | 2(0.369) |
| ERR221592 | single | 1 | 2(0.25) | 2(0.047) | 2(0.478) |
| ERR221611 | both | 0.87 | 3(0.102) | 2(0.201) | 2(0.357) |
| ERR245716 | single | 1 | 2(0.279) | 2(0.099) | 2(0.29) |
| ERR245754 | both | 0.57 | 3(0.093) | 2(0.064) | 2(0.007) |
| ERR245758 | both | 0.79 | 2(0.078) | 2(0.122) | 2(0.038) |
| ERR245795 | both | 0.65 | 2(0.112) | 2(0.196) | 2(0.147) |
| ERR245797 | both | 0.82 | 2(0.07) | 2(0.085) | 2(0.054) |
| ERR323044 | both | 0.71 | 2(0.052) | 2(0.272) | 2(0.208) |
| ERR323054 | both | 0.66 | 5(0.192) | 2(0.323) | 2(0.152) |
| ERR323056 | single | 1 | 4(0.348) | 2(0.481) | 2(0.484) |
| ERR323082 | both | 0.71 | 4(0.114) | 2(0.206) | 2(0.162) |
| ERR473322 | both | 0.77 | 4(0.195) | 2(0.213) | 2(0.237) |
| ERR473340 | single | 1 | 5(0.38) | 2(0.043) | 2(0.437) |
| ERR473359 | both | 0.5 | 3(0.063) | 2(0.234) | 2(0.017) |
| ERR773806 | both | 0.91 | 4(0.3) | 2(0.19) | 2(0.353) |
